# Effect of Training-Induced Changes in Achilles Tendon Stiffness on Muscle–Tendon Behavior During Landing

**DOI:** 10.3389/fphys.2018.00794

**Published:** 2018-06-26

**Authors:** Amelie Werkhausen, Kirsten Albracht, Neil J. Cronin, Gøran Paulsen, Jens Bojsen-Møller, Olivier R. Seynnes

**Affiliations:** ^1^Department of Physical Performance, Norwegian School of Sport Sciences, Oslo, Norway; ^2^Institute of Biomechanics and Orthopaedics, German Sport University Cologne, Cologne, Germany; ^3^Department of Medical Engineering and Technomathematics, Aachen University of Applied Sciences, Aachen, Germany; ^4^Neuromuscular Research Centre, Faculty of Sport and Health Sciences, University of Jyväskylä, Jyväskylä, Finland; ^5^The Norwegian Olympic and Paralympic Committee and Confederation of Sports, Oslo, Norway

**Keywords:** Achilles tendon, energy absorption, mechanical buffer, stiffness, tendon properties, energy dissipation

## Abstract

During rapid deceleration of the body, tendons buffer part of the elongation of the muscle–tendon unit (MTU), enabling safe energy dissipation via eccentric muscle contraction. Yet, the influence of changes in tendon stiffness within the physiological range upon these lengthening contractions is unknown. This study aimed to examine the effect of training-induced stiffening of the Achilles tendon on triceps surae muscle–tendon behavior during a landing task. Twenty-one male subjects were assigned to either a 10-week resistance-training program consisting of single-leg isometric plantarflexion (*n* = 11) or to a non-training control group (*n* = 10). Before and after the training period, plantarflexion force, peak Achilles tendon strain and stiffness were measured during isometric contractions, using a combination of dynamometry, ultrasound and kinematics data. Additionally, testing included a step-landing task, during which joint mechanics and lengths of gastrocnemius and soleus fascicles, Achilles tendon, and MTU were determined using synchronized ultrasound, kinematics and kinetics data collection. After training, plantarflexion strength and Achilles tendon stiffness increased (15 and 18%, respectively), and tendon strain during landing remained similar. Likewise, lengthening and negative work produced by the gastrocnemius MTU did not change detectably. However, in the training group, gastrocnemius fascicle length was offset (8%) to a longer length at touch down and, surprisingly, fascicle lengthening and velocity were reduced by 27 and 21%, respectively. These changes were not observed for soleus fascicles when accounting for variation in task execution between tests. These results indicate that a training-induced increase in tendon stiffness does not noticeably affect the buffering action of the tendon when the MTU is rapidly stretched. Reductions in gastrocnemius fascicle lengthening and lengthening velocity during landing occurred independently from tendon strain. Future studies are required to provide insight into the mechanisms underpinning these observations and their influence on energy dissipation.

## Introduction

Elastic properties of tendons are inextricably linked to the mechanical output of the muscle to which they are attached. The function of elastic tissue and energy storage includes but is not limited to the conservation of energy or the power amplification observed during locomotor tasks (Roberts and Azizi, 2011). As shown in animals (Roberts and Azizi, 2010; Roberts and Konow, 2013) and in humans (Werkhausen et al., 2017), tendons also act as mechanical buffers to accommodate rapid stretches of the muscle–tendon unit (MTU) and thus contribute to mechanical energy dissipation via lengthening contractions. The buffering function of the tendon provides a controlled means for the MTU to absorb energy, and is associated with a mechanism to protect muscle fascicles against damage caused by rapid and excessive strain (for review see Roberts and Konow, 2013). Thus, during a task where power attenuation is required, the tendon mechanical properties affect the active lengthening of muscle fibers and their ability to dissipate energy. It follows that changes in tendon properties may affect muscle–tendon interaction and energy dissipation in the MTU.

There is a consensus among studies regarding tendon’s adaptability to mechanical loading (Wiesinger et al., 2015). In humans, several studies have demonstrated a stiffening of the Achilles tendon after various types of resistance training (e.g., Kubo et al., 2002; Arampatzis et al., 2007). Additionally, tendinous adaptations seem to occur in parallel with muscular adaptations (Miller et al., 2005; Seynnes et al., 2009), suggesting the influence of the former on work production during locomotor activities.

Modeling studies have demonstrated the theoretical link between locomotion efficiency, tendon stiffness and muscle architecture. Simulations using Hill-type muscle models have shown that during walking and running, contractile efficiency is maximized at optimal combinations of tendon compliance and muscle fascicle length (Lichtwark and Wilson, 2007, 2008). Accordingly, a recent study reported that muscle–tendon behavior during a stretch-shortening exercise was altered after a training intervention that increased Achilles tendon stiffness (Hirayama et al., 2017). Hirayama et al. (2017) interpreted these findings as an optimisation of the muscle–tendon behavior to produce power, via a decrease in fascicle shortening velocity and an increase in tendon shortening velocity. A similar relation between changes in tendon compliance and muscle contractile behavior may apply to lengthening contractions in energy dissipation tasks, but this question has not been investigated to date.

Hence, the aim of this study was to investigate the effect of a resistance training-induced increase in Achilles tendon stiffness on the behavior of the triceps surae MTU during a task requiring energy dissipation. We predicted that training-induced stiffening of the Achilles tendon would reduce tendon strain during the landing task. This decrease was in turn expected to reduce the buffering action of the tendon and increase the magnitude and velocity of fascicle lengthening.

## Materials and Methods

### Experimental Protocol and Participants

Twenty-one recreationally active volunteers provided written informed consent to participate in the present study, which was approved by the ethical committee of the Norwegian School of Sport Sciences. *A priori* power calculations based on a previously observed increase in Achilles tendon stiffness with isometric training (Arampatzis et al., 2007) suggested a power of 0.96 with a sample size of *n* = 8. Eleven subjects were assigned to the training group (height 174 ± 9 cm, body mass 70 ± 9 and 69 ± 9 kg before and after training, respectively, age 26 ± 4 years, five men and six women). Exclusion criteria were musculoskeletal disorders preventing the possibility to perform resistance training, and regular strength training of the plantar flexor muscles prior to the study. The subjects in the training group underwent a regimen of explosive, isometric plantarflexions for 10 weeks, while the remaining ten subjects (height 178 ± 8 cm, body mass 73 ± 8 kg before and after training, age 30 ± 3 years, six men and four women) were assigned to the control group. Control subjects did not engage in any resistance training for the plantar flexors and were instructed not to change their daily activities during the course of the study. All participants went through the same protocol before and after the training period where (1) anthropometrics and mechanical properties of the triceps surae MTU were measured, and (2) muscle–tendon behavior during an energy dissipation task was examined by use of ultrasonography, kinematics and kinetics analyses. Before testing subjects performed a warm-up exercise consisting of 5-min running on a treadmill at self-selected speed. For the energy dissipation task, subjects were asked to perform single-leg drop landings from a height of 15 cm. This height was chosen to allow landing with minimal knee flexion. All measurements were performed on the right leg.

### Exercise Program

The training group performed standing isometric unilateral plantarflexions three times per week for 10 weeks. Each training session started with a 5-min warm up on a cycle ergometer, followed by four sets of ten explosive contractions (1 s loading, 5 s rest). Explosive contractions with a short time under tension were used in an attempt to minimize strength gains and muscle hypertrophy (Cormie et al., 2010; Balshaw et al., 2016) while eliciting a stiffening of the Achilles tendon. For the training task, subjects stood on one leg in an adjustable and rigid custom-built rig with their ankle and knee joints in anatomical position, facing a wall. The position of horizontal shoulder arms attached to the wall was adjusted to the shoulder height of each individual. A taut cable that ran vertically between the shoulder arms and the ground prevented upward displacement of the subjects’ shoulders, enabling isometric plantar flexion contractions. Strength measurements used to monitor and adjust training load were obtained from a strain gauge placed in series with the vertical cable. Subjects were instructed to reach the target force as fast as possible. The contraction intensity was set to 80% of maximum force and was adjusted during the first training session of every week. Pilot testing indicated that this force level was the highest that subjects could reach reliably across four sets of 10 repetitions. Visual feedback of instantaneous force was provided to the subjects to enable them to match the target force during training and to visualize maximal force during testing.

### Properties of the Muscle–Tendon Unit

#### Muscle Architecture

Ultrasound images of resting muscle architecture were taken from the mid-belly of the muscle (i.e., mid-length between the popliteal fossa and the gastrocnemius myotendinous junction and along the mediolateral width) when the subjects were lying prone with the hip, knee, and ankle joints in anatomical position (HL9.0/60/128Z-2, LS 128 Telemed, Vilnius, Lithuania). Fascicle length, pennation angle and thickness of gastrocnemius medialis were measured offline with software for image analysis (ImageJ, National Institutes of Health, Bethesda, MD, United States). Fascicle length was measured as a straight line aligned with visible portions of fascicles, between the superficial and deep aponeuroses. A linear extrapolation was necessary in the few cases when the fascicles did not fit within the 60 mm field of view. Pennation angle was defined as the angle between the segmented fascicle and the orientation of the deep aponeurosis. Muscle thickness was calculated as the average of the shortest distances between the two aponeuroses, measured at 25, 50, and 75% of the width of the field of view.

#### Maximal Voluntary Torque

Subjects lay prone on a dynamometer (IsoMed 2000 D. & R. Ferstl GmbH, Hemau, Germany) with hip, knee, and ankle joints firmly fixed at anatomical positions. Stiff pads and straps were adjusted to restrict the movement of the trunk and knee joint. The axis of the dynamometer was carefully aligned with the rotation axis of the ankle joint, and foot straps, knee and shoulder pads were adjusted to minimize movement. As a specific warm up, subjects performed at least five submaximal contractions of the plantar flexors. The maximal plantarflexion torque was determined as the highest of at least two maximal voluntary contractions.

#### Achilles Tendon Mechanical Properties

In the same position, ultrasound scans (80 Hz) of the gastrocnemius myotendinous junction, plantarflexion torque (600 Hz) and kinematic marker trajectories (120 Hz) were recorded simultaneously during ramp contractions to estimate tendon stiffness. The ultrasound transducer was fixed with adhesive tape over the myotendinous junction of the gastrocnemius. A gel pad was positioned between the skin and the transducer to allow consistent scanning when the muscle was bulging. Four motion analysis cameras (Qualisys, Gothenburg, Sweden) captured the positions of three reflective markers attached to the ultrasound probe and one marker on the calcaneus over the tendon insertion. A trigger signal from the ultrasound system synchronized all measurements. Subjects were provided with visual feedback and instructed to exert a torque at a loading rate of 100 N m s^-1^ up to 90% of the individual maximal torque. Ramp contractions at constant loading rate were performed before trials were recorded, to familiarize the subjects with the task and to ensure preconditioning of the tendon (Maganaris, 2003b).

Marker trajectories were filtered with a second order bidirectional low-pass Butterworth filter with a cut-off frequency of 15 Hz. The position of the gastrocnemius myotendinous junction was tracked offline semi-automatically by following the closest visible fascicle insertion (Tracker 4.95 ^1^). The fascicle insertion was chosen in a region 1–3 cm proximal to the actual muscle–tendon junction. The distance between the tracked features and the muscle–tendon junction was constant in all the video scans of each subject. Ultrasound data were filtered with a second order bidirectional low-pass Butterworth filter with a cut-off frequency of 6 Hz. Prior calibration established the position of the ultrasound image relative to the kinematic markers on the cast used to hold the ultrasound transducer. This enabled calculation of the position of the myotendinous junction in the laboratory coordinates system. Thus, Achilles tendon length was calculated as the distance between the myotendinous junction and the calcaneus marker during the contraction (Gerus et al., 2011). Plantarflexion torque data was filtered, similarly to the kinematic data, and the Achilles tendon force was estimated by dividing the torque by the internal moment arm, measured externally with a tape measure as the mean perpendicular distance from the tendon to the midpoint between the medial and lateral malleolus. For further analysis, 91% of the calculated tendon force was used to represent the proportion of total ankle moment attributable to the triceps surae (Dick et al., 2016). Using kinematics, the torque was corrected to account for inevitable ankle joint rotation (Arampatzis et al., 2005) and gravitational forces (Karamanidis et al., 2005). Subsequently, the tendon force-elongation plots of three out of five trials, where the trials with the highest and lowest stiffness were excluded, were averaged and fitted with a third order polynomial. Tendon stiffness was calculated for every subject as the slope of the fitted force-elongation curve between 50 and 80% of the maximum individual force level, and maximum tendon strain was measured at the maximum common force of pre- and post-tests for every subject.

### Mechanics of the Landing Task

#### Joint Mechanics

Ankle and knee joint mechanics were measured from the right leg during single-leg landings. Twenty retroreflective markers were attached to the skin and captured by at least 12 cameras operating at 300 Hz. Motion capture was synchronized with force plates recording at 1500 Hz (Force-Link, Motek, Netherlands). A modified Cleveland Clinics marker set (left and right anterior and posterior iliac spine; right medial and lateral epicondyles; right medial and lateral malleoli; posterior calcaneus and first, second, and fifth metatarsal; two clusters of four markers to track the right thigh and shank segment, respectively) was used to calculate ankle and knee joint angles, moments and powers (Visual 3D, C-Motion, Germantown, MD, United States). For the purpose of the analysis, the landing phase was defined as the phase of negative ankle power production. Ground reaction force (GRF) and trajectory data were filtered at 15 Hz using a bidirectional second order low-pass Butterworth filter. Gastrocnemius and soleus MTU lengths were estimated from ankle and knee joint angles and shank length data (Hawkins and Hull, 1990). Shank length was measured externally, from the lateral malleolus to the lateral epicondyle.

#### Tendon Mechanics

Achilles tendon length during landing was estimated using the same procedure as that described above for the measurement of tendon stiffness, with the same frame rate of 80 Hz. The instantaneous Achilles tendon moment arm was calculated as the shortest perpendicular distance from the tendon to the ankle joint center defined as the midpoint of the malleoli markers (Obst et al., 2017). Achilles tendon force was then estimated by dividing ankle joint moment by the moment arm (Fukashiro et al., 1993).

#### Fascicle Mechanics

Ultrasound videos were recorded in additional trials to examine changes in gastrocnemius and soleus fascicle length. The use of ultrasonography to determine muscle fascicle length has previously been shown to provide satisfactory levels of reliability (Gillett et al., 2013; Kwah et al., 2013). The transducer was positioned over the gastrocnemius muscle belly, so that soleus fascicles were also visible. A transducer cast and self-adhesive tape prevented movement of the transducer. Fascicle lengths and pennation angles were obtained using a semi-automated tracking algorithm (Cronin et al., 2011; Farris and Lichtwark, 2016). Ultrasound images from each group were analyzed in a random order (pre-post) by the same investigator. Fascicle, tendon, and MTU velocities were obtained by differentiating the corresponding length with respect to time.

### Data Reduction and Statistical Analysis

Analysis of changes in variables of interest during the landing task was performed over the period of negative power production at the ankle joint, to reflect the duration of energy absorption and dissipation. All time series data were resampled over 101 points and averaged across 4–5 trials per subject. Individual mean time series were subsequently averaged across subjects for each group. A repeated-measures, two-way ANOVA with the factors time of testing (pre- vs. post training) and group (training group vs. control group) was used to test differences in Achilles tendon properties, muscle architecture and muscle–tendon behavior at relevant phases of the landing task. Sidak *post hoc* tests were employed in case of significant main effects or time by group interactions. Pearson correlation coefficients were calculated between the changes in tendon stiffness and changes in tendon strain and fascicle lengthening during landing. Statistical significance was set to *P* < 0.05. Results are presented as mean ± standard deviation in the text and as mean ± standard error of the mean in the line figures to illustrate the precision of the mean.

## Results

### Muscle–Tendon Properties and Maximal Strength

**Table 1** shows the changes in muscle–tendon properties of both groups after the training period. The one repetition maximum of the training group increased from 1670 ± 393 N in week one to 2317 ± 607 N in week 10. Maximal isometric plantarflexion torque increased on average by 15%, with concomitant 5% increases in gastrocnemius pennation angle and muscle thickness, without any change in fascicle length. Achilles tendon stiffness measured before and after training increased on average by 18%, although the strain at the individualized maximal force reached in pre- and post-intervention tests (1683 ± 463 N) did not decrease significantly (mean change: -9%, mean ± SD: 3.4 ± 0.7 and 3.1 ± 0.5%). Individual and mean force-elongation relationship of the Achilles tendon are presented in the Supplementary Figure S1. None of these variables changed significantly in the control group.

**Table 1:** Plantarflexion strength (torque), resting muscle architecture and Achilles tendon (AT) stiffness in the training group (TG) and the control group (CG) measured in the pre-test (pre) and the post-test (post).

	TG			CG		
	Pre	Post	CI	Pre	Post	CI
Torque [N m]	172 ± 50	198 ± 51	-11 to -42^∗^	170 ± 51	180 ± 62	7 to -27
L_f_ GM [mm]	892 ± 133	907 ± 161	42 to -72	842 ± 161	840 ± 104	62 to -59
PA GM [deg]	18.1 ± 1.8	19.0 ± 2.0	0 to -1.7^∗^	18.4 ± 1.1	18.3 ± 1.7	1 to -0.8
Thickness GM [mm]	237 ± 35	250 ± 38	0 to -27^∗^	231 ± 38	235 ± 36	9 to -18
AT stiffness [N mm^-1^]	397 ± 146	459 ± 147	-14 to -109^∗^	399 ± 193	400 ± 212	48 to -51
AT strain [mm]	4.4 ± 1.1	4.1 ± 0.5	-0.3 to 1.1	4.0 ± 1.5	3.9 ± 1.5	-0.6 to 0.8

*Values are means ± SD; CI, 95% confidence interval of differences; GM, gastrocnemius medialis; SOL, soleus; L_*f*_, length of fascicle; PA, pennation angle; ^∗^P < 0.05 comparing pre and post-test*.

### Kinematics and Kinetics During the Landing Task

**FIGURE 1:**
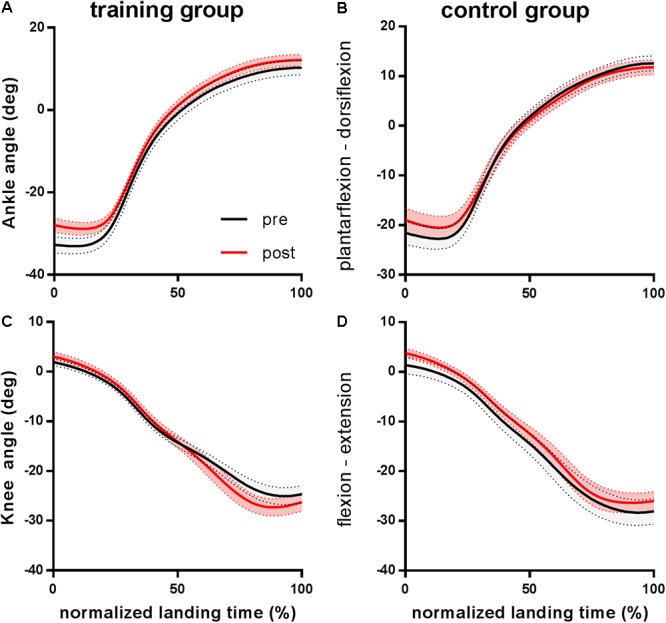
Group mean ankle **(A,B)** and knee joint **(C,D)** angles during energy absorption in the training group and the control group before (pre) and after (post) the training period. Time series are normalized to 101 points. Negative changes in angles correspond to ankle plantarflexion and knee flexion.

Subjects in both groups executed the landing task in the same way at pre- and post-training, as evidenced by similar landing duration, peak GRF, knee and ankle joint moment and power, and Achilles tendon force (**Table 2**). While control subjects landed in a less plantar flexed position than trained subjects (*P* = 0.001), in the training group the ankle angle at touch down was less plantar flexed after training (*P* = 0.026). While no interaction effect was found for the ankle angle at touch down, a group effect showed that control subjects landed in a less plantar flexed position than trained subjects (*P* = 0.001). The analysis also showed a main effect for time, with *post hoc* tests indicating a less plantar flexed ankle angle at touchdown after training for the training group (*P* = 0.026) but not for the control group (*P* = 0.306). The excursion of the ankle joint during the task did not change significantly after the intervention, as evidenced by a lack of significant group × time interaction effect (*P* = 0.937). A trend toward a reduced plantarflexion during landing was observed in post-tests (-6% in the training group and -7% in the control group) but did not reach significance (*P* = 0.110). Knee joint angle at touchdown and knee joint excursion were statistically similar between groups and after training (**Figure 1**).

**Table 2:** Peak ankle and knee moment and power, Achilles tendon (AT) force, MTU work and landing duration in the training group (TG) and the control group (CG) measured in the pre-test (pre) and the post-test (post).

	TG		CG	
	Pre	Post	Pre	Post
Ankle moment [N m]	-143 ± 34	-141 ± 30	-153 ± 26	-154 ± 34
Ankle power [W]	-976 ± 260	-1012 ± 186	-1027 ± 192	-998 ± 193
Ankle work [J]	-78 ± 25	-70 ± 30	-74 ± 21	-69 ± 13
Knee moment [N m]	90 ± 45	108 ± 42	117 ± 45	112 ± 40
Knee power [W]	-314 ± 203	-455 ± 218	-549 ± 245	-532 ± 312
Knee work [J]	-16 ± 16	-19 ± 13	-27 ± 15	-20 ± 16
AT force [N]	2746 ± 555	2750 ± 518	2848 ± 493	2857 ± 736
MTU work [J]	40 ± 13	39 ± 10	34 ± 8	30 ± 7
Duration [s]	0.205 ± 0.014	0.202 ± 0.013	0.204 ± 0.020	0.202 ± 0.020

*Values are means ± SD; AT, Achilles tendon, no statistical differences were observed between pre- and post-test*.

### Muscle–Tendon Measures During the Landing Task

**FIGURE 2:**
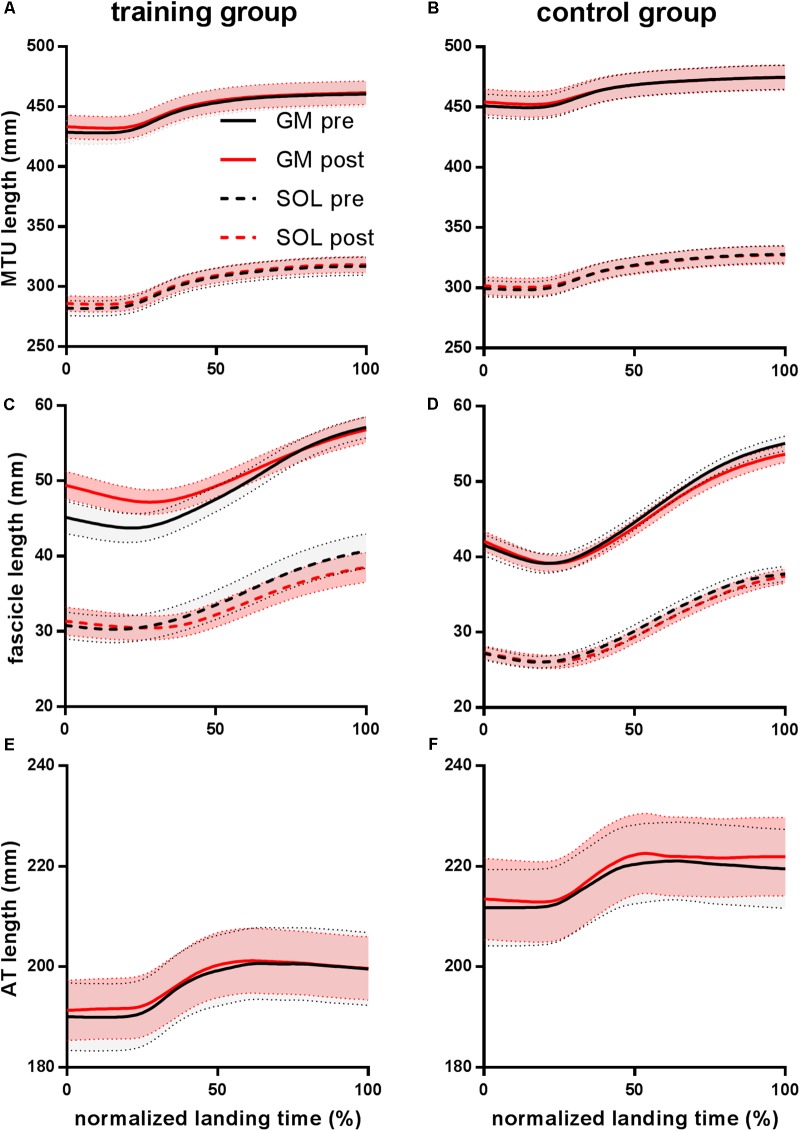
Muscle–tendon unit (MTU) **(A,B)** and fascicle **(C,D)** lengths of gastrocnemius medialis (GM) and soleus (SOL), and Achilles tendon (AT) length **(E,F)**. Time series are normalized to 101 points.

The mean changes in length of MTUs and their components (fascicles, Achilles tendon only, and muscle) during landing are presented for each group, before and after training, in **Figure 2**. After an initial isometric phase (≈20% of landing duration), soleus and gastrocnemius MTUs lengthened until the end of the landing period. During the first half of the landing, the Achilles tendon stretched, similarly to the whole MTU and did not lengthen further in the second half. In contrast, muscle fascicles initially shortened (*P* < 0.001), before lengthening throughout the rest of the landing period (*P* < 0.001). In line with the differences in ankle joint angle mentioned above, group differences in length of MTU were observed at touch down. In addition, gastrocnemius – but not soleus – muscle and fascicles lengthened more during landing in the training group than in controls before the intervention (**Table 3**).

**Table 3:** Peak length changes and velocities of the muscle–tendon unit (MTU), muscle fascicles, elastic element (EE), Achilles tendon (AT), and muscle length during landing in the training group (TG) and the control group (CG) measured during pre-test (pre) and post-test (post).

		TG			CG		
		Pre	Post	CI	Pre	Post	CI
Δl Max lengthening [mm]	MTU GM α	-32 ± 7	-30 ± 6	-7.1 to 1.4	-25 ± 6	-23 ± 6	-6.9 to 2.0
	MTU SOL	-35 ± 7	-33 ± 5	-6.3 to 2.0	-30 ± 6	-27 ± 6	-6.7 to 2.1
	Fascicles GM α	-14 ± 4	-10 ± 2	-6.1 to -1.1^∗^x	-16 ± 3	-15 ± 2	-4.0 to 1.1
	Fascicles SOL	-11 ± 4	-8 ± 2	-4.2 to -0.2^∗^	-12 ± 3	-11 ± 2	-2.4 to 1.8
	AT GM	-12 ± 2	-11 ± 3	-2.3 to 1.0	-10 ± 3	-11 ± 3	-1.0 to 2.6
	Muscle GM	-23 ± 7	-22 ± 6	-5.5 to 3.4	-18 ± 7	-15 ± 7	-7.4 to 1.8
Peak vel [mm s^-1^]	MTU GM	617 ± 163	560 ± 118	-41 to 155	508 ± 131	451 ± 132	-46 to 160
	MTU SOL	610 ± 151	567 ± 100	-47 to 134	514 ± 115	467 ± 114	-47 to 142
	fascicles GM	134 ± 44	106 ± 26	0 to 56^∗^x	155 ± 24	148 ± 24	-23 to 36
	fascicles SOL	102 ± 26	91 ± 17	-9 to 30	118 ± 32	112 ± 19	-15 to 25
	AT GM	265 ± 36	250 ± 58	-24 to 55	264 ± 73	297 ± 88	-74 to 9

*Values are means ± SD; CI, 95% confidence interval of differences; GM, gastrocnemius medialis; SOL, soleus; ^∗^P < 0.05 comparing raw values pre- and post-test, §P < 0.05 comparing values normalized to instantaneous MTU length pre- and post-test, bbb[scale=0.4]img001 P < 0.05 comparing baseline, raw values between the groups. NB: When normalized to MTU length, all variables were similar between groups at baseline*.

At the beginning of the negative power phase, gastrocnemius fascicles were longer for the training group after training (*P* = 0.012) whereas this variable did not change for the control group (*P* = 0.930). Soleus fascicle length at this time point was similar before and after training (*P* = 0.685). Consistent with fascicle length, gastrocnemius pennation angle at touchdown was lower after training (*P* = 0.035), while tendon length did not change (*P* = 0.707).

**FIGURE 3:**
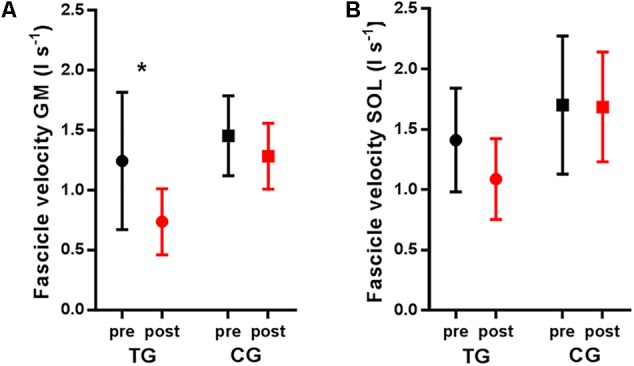
Average fascicle velocity in the training group (TG) and the control group (CG) measured during pre-test (pre) and post-test (post). **(A)** Gastrocnemius medialis (GM) and **(B)** soleus (SOL) fascicle velocities were calculated as the first derivative of the normalized fascicle length. Values are means ± SD. ^∗^*P* < 0.05 comparing pre- and post-test.

**FIGURE 4:**
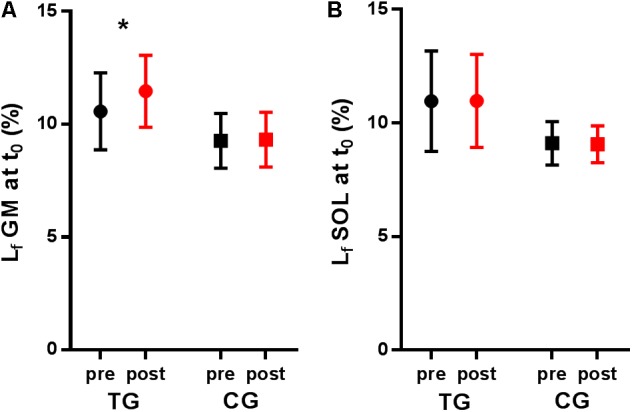
Fascicle length at touch down in the training group (TG) and the control group (CG) measured during pre-test (pre) and post-test (post). **(A)** Gastrocnemius medialis (GM) and **(B)** soleus (SOL) fascicle lengthening were normalized to the respective MTU lengths. ^∗^*P* < 0.05 comparing pre- and post-test.

After the training intervention, the onset of fascicle lengthening occurred later for the training group (+7% of the landing period for the gastrocnemius, *P* = 0.002 and +8% for the soleus, *P* = 0.014, respectively) but not for the control group (+1%, *P* = 0.788 and +3%, *P* = 0.614, respectively). A significant effect of training was also seen on the magnitude of gastrocnemius and soleus fascicle lengthening. *Post hoc* tests showed that fascicle lengthening of both muscles decreased in the training group only (**Table 3** and **Figure 2**). Accordingly, peak shortening and mean shortening velocity of the gastrocnemius fascicles were significantly reduced in the trained group, although this reduction did not reach significance for soleus (**Table 3** and **Figure 3**). To ensure that the observed effects of training on fascicle length and velocity were not due to slight differences in the execution of the task between groups and time points, the same statistical analysis was performed on fascicle length values normalized to instantaneous MTU length. This additional analysis confirmed the differences described in **Table 3** and **Figure 4**. Neither fascicle shortening nor lengthening of MTU and tendon differed significantly after the training period (**Table 3**). No significant correlations were found between the change in stiffness and tendon strain or fascicle lengthening (*r* = -0.055 and *P* = 0.814, *r* = -0.373 and *P* = 0.096).

## Discussion

The aim of this study was to examine the effects of a training-induced increase in Achilles tendon stiffness on muscle–tendon mechanics when power attenuation is required, during a drop landing task. Our main hypothesis was that a stiffer tendon would less effectively buffer the lengthening of muscle fascicles against MTU lengthening. Before training, the overall behavior of muscle fascicles and elastic structures during the landing task was consistent with previous observations, showing the buffering function of elastic structures to limit the amplitude and velocity of fascicle lengthening (Roberts and Azizi, 2010; Werkhausen et al., 2017). The training intervention increased Achilles tendon stiffness (18%) and muscle strength (15%). Contrary to our predictions, these adaptations did not change the elongation of the Achilles tendon but were accompanied by a reduction in gastrocnemius fascicle lengthening during the negative ankle power phase. Despite reduced fascicle lengthening and unchanged joints moment, the same amount of negative work was done at the ankle and the knee after training. These results support the notion that altered tendon properties can influence the contractile behavior of the triceps surae muscles during a landing task, although the impact of these changes on energy flow remains elusive.

### Changes in Muscle–Tendon Properties

Training-induced changes in muscle architecture vary considerably between reports and may depend on training modalities (i.e., contraction type and velocity, (for review see Timmins et al., 2016). Relatively small but significant increases in pennation angle and muscle thickness were measured in the present study. These adaptations are typically associated with additional sarcomeres in parallel and are consistent with the observed increase in plantarflexion strength. However, the fascicle length was not affected by the training protocol.

The 18% increase in tendon stiffness induced by the present protocol is consistent with results from previous interventions based on isometric contractions (Arampatzis et al., 2010; Albracht and Arampatzis, 2013). Although a reduction in strain measured during isometric contractions was expected with tendon stiffening, this variable did not change significantly in the present study, which is nevertheless consistent with some previous reports (e.g., Arampatzis et al., 2007). This apparent discrepancy may be attributable to the lack of sensitivity of tendon strain measurements and the difficulty to assess tendon slack length *in vivo* (Seynnes et al., 2015). Additionally, while we were able to test the Achilles tendon, changes in the mechanical properties of other elastic elements (i.e., aponeuroses, connective tissue, and proximal tendon) could not be assessed, and it is speculated that other collagen-based structures may also have become stiffer as the result of the intervention. These possibilities can unfortunately not be verified here, and we conclude that tendon stiffening was insufficient to reduce Achilles tendon strain significantly.

### Landing Kinematics

The more dorsiflexed position at the beginning of the landing in the training group and the same trend in the control group suggests that a slight change in the kinematic strategy occurred between pre- and post-tests. Despite the practice trials included in our protocol, this trend is possibly attributable to an improvement of the task execution. Alternatively, unaccounted effects of the training intervention (e.g., change in optimal angle of torque production, increase in rate of force development) may have led the trained subjects to land in a less plantarflexed position after training. However, since no significant differences were found between pre- post-training values of joint angle excursion and MTU lengthening during the landing period, the execution of the landing task was similar overall before and after training. Furthermore, the similarity between raw and normalized (i.e., to MTU length) strain data confirms that the observed changes after training were not entirely due to variability in the execution of the task.

### Muscle and Tendon Mechanics During Landing

The unaltered tendon strain measured during isometric contractions after training is congruent with the similar tendon elongation observed during landing, despite unchanged joint moments. As mentioned above (see *Changes in muscle–tendon properties*), a type II error cannot be entirely ruled out, but an insufficient increase in stiffness to constrain tendon strain seems a more likely explanation. It can be speculated that the increase in stiffness (<100 Nmm^-1^) found in this study was not sufficient to substantially reduce tendon longitudinal strain during landing. Alternatively, recent studies have highlighted the complexity of tendon deformation (Farris et al., 2013; Raiteri et al., 2016), and in particular the two-dimensional nature of this deformation in the proximal part of the Achilles tendon. The present intervention may have resulted in direction-specific changes in tendon mechanical properties, limiting deformation in the transverse direction. Likewise, the structural changes of the muscle due to hypertrophy may have resulted in different two-dimensional strain patterns in the Achilles tendon. Future studies looking at the links between tendon properties and function should include transverse strain measurements to verify these hypotheses.

Despite the lack of significant change in longitudinal deformation of the tendon, the active lengthening of the gastrocnemius fascicles during landing was reduced after training. A similar trend was found in soleus, but this disappeared after accounting for MTU length changes, suggesting distinct roles of the two muscles or a smaller effect of training on soleus. Here again, region-specific changes in tendon strain patterns or mechanical properties may have limited the effect of training on the free tendon and therefore the soleus contractile behavior. In support of this possibility, recently published modeling data demonstrate the reduced sensitivity of soleus muscle–tendon mechanics to changes in tendon compliance compared to gastrocnemius (Orselli et al., 2017). Consistent with the data on fascicle lengthening during landing, gastrocnemius fascicle lengthening velocity was also reduced after training. A slower active lengthening of the fascicles does not provide any advantage for force production, but may mitigate the damage caused by faster eccentric contractions (Proske and Morgan, 2001).

In the case of the gastrocnemius muscle, the offset toward longer fascicle length at touch down is noteworthy. Initial fascicle length may have conditioned lengthening magnitude if this parameter was determined by the final standing position (i.e., at the end of the landing phase). Owing to the gastrocnemius force-length relationship, the increased fascicle length may have increased the force produced at a similar level of muscle activation, which could partly explain the reduction in fascicle lengthening after training. Another possible reason for this offset is the variability in fascicle segmentation on the first image of each ultrasound scanning sequence. A recent analysis of the reliability of fascicle tracking (Aeles et al., 2017) found good reliability for the analysis of fascicle length changes but also pointed at the more variable segmentation of the initial image, rendering the comparison of absolute lengths more challenging. However, in the present study, the same investigator (AW) analyzed all images in a random order and the fact that no differences in fascicle length at touch down were found in the control group support these findings. So, is this offset induced by training or simply linked to the reduction in ankle plantarflexion at touchdown? The latter interpretation seems unlikely here, because the same analyses performed on the values normalized to instantaneous MTU length confirmed that fascicles were longer at the beginning of the landing (**Figure 4**). Since training did not induce any change in resting fascicle length, a possible explanation for the differences seen at touch down could lie in the stiffening of elastic structures proximal to the Achilles tendon (i.e., proximal tendon, aponeurosis, connective tissue), which would constrain fascicles to a longer length at touch down. This hypothesis would also be consistent with the delayed onset of fascicle lengthening after training, because of an increased resistance of elastic tissue. It would additionally be compatible with the fact that the offset was seen for the gastrocnemius but not for the soleus muscle.

### Energy Storage and Dissipation

The fact that the task and the mass of the subjects were identical before and after the intervention implies that requirements for energy storage and dissipation were unchanged. However, in the trained subjects, the increased plantarflexion strength combined with a stiffer Achilles tendon that underwent the same elongation after training, indicates that more energy was being stored during landing. Had fascicles produced more force, a similar amount of energy would have been dissipated despite a reduced lengthening. Increased fascicle force production would have been consistent with the observed muscle hypertrophy. It would also be in line with the longer operating length of the fascicles after training, since the triceps surae muscles operate on the ascending limb of the force-length relationship (Maganaris, 2003a). However, such an increase is incompatible with our calculations of tendon force during landing, which did not change after training. This point and the unaltered work done at the ankle and knee joints make the interpretation of the findings difficult in relation to the energetic consequences of the observed changes. Of course, the accuracy of the estimated tendon force is limited by a number of assumptions, amongst which antagonist co-activation level was similar between subjects before and after the intervention. Despite the limited predictive value of electromyographic amplitude to estimate antagonist torque, future studies may use other *in vivo* solutions to improve force estimation. For the time being, the unaltered Achilles tendon force and work done at the ankle and knee joints make the interpretation of the findings difficult in relation to the energetic consequences of the observed changes.

## Conclusion

This study showed that training-induced increases in Achilles tendon stiffness and muscle strength did not affect the buffering capacity of the tendon against large fascicle strain during landing. On the contrary, decreases in gastrocnemius fascicle lengthening and lengthening velocity were observed after training, although it could not be directly linked to unchanged longitudinal strain of the tendon. Questions remain regarding the mechanisms underpinning the observed changes in fascicle behavior, and further research is warranted to assess more precisely the impact of these changes on energy dissipation. The techniques currently used to study muscle–tendon mechanics *in vivo* are limited by necessary assumptions and possible associated errors. Reducing the likelihood and effects of these errors will allow the present findings to be confirmed and built upon.

## Ethics Statement

This study was carried out in accordance with the recommendations of the institutional ethical committee, Ethical Committee of the Norwegian School of Sport Sciences. The protocol was approved by the Ethical Committee of the Norwegian School of Sport Sciences. All subjects gave written informed consent in accordance with the Declaration of Helsinki.

## Author Contributions

AW and OS performed the experiments, analyzed the data, and drafted the manuscript. AW, KA, NC, GP, JB-M, and OS were all involved in the conception and design of the research and edited, revised, and approved the final version of the manuscript.

## Conflict of Interest Statement

The authors declare that the research was conducted in the absence of any commercial or financial relationships that could be construed as a potential conflict of interest.

## Footnotes

1 physlets.org/tracker/
