# Supplementary material for: Effect of Training-Induced Changes in Achilles Tendon Stiffness on Muscle–Tendon Behavior During Landing
Source: Front Physiol. 2018 Jun 26;9:794. doi: 10.3389/fphys.2018.00794 (PMC6028711; doi:10.3389/fphys.2018.00794)
Supplement: Supplementary file 1 [file Image_1.PDF]

Supplementary

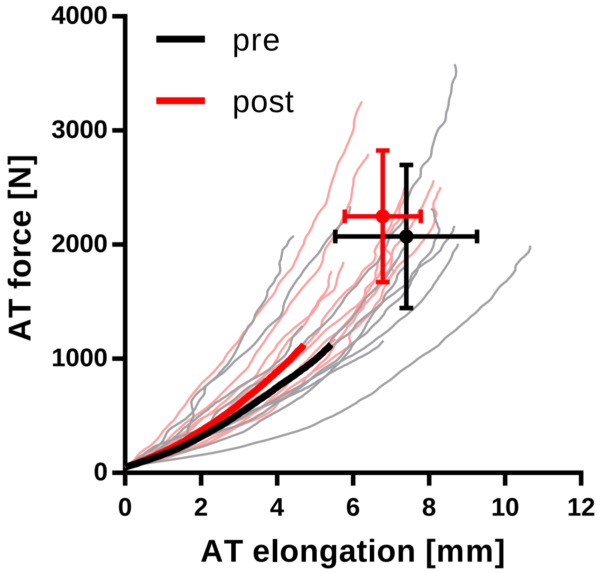

Figure 1. Force-elongation relationship of the Achilles tendon of the training group pre and post training. Bold lines and dots indicate mean valus, thin lines represent individual data from. The mean increase in tendon stiffness was 18% (*P* = 0.0009). Note that the individual curve within the lowest force range is outside the mean force range of other curves but the increase in stiffness remains significant (+18%, *P* = 0.0014) without the data from this subject.
